# Association of Carotid Plaque Morphology and Glycemic and Lipid Parameters in the Northern Manhattan Study

**DOI:** 10.3389/fcvm.2022.793755

**Published:** 2022-01-24

**Authors:** David Della-Morte, Chuanhui Dong, Milita Crisby, Hannah Gardener, Digna Cabral, Mitchell S. V. Elkind, Jose Gutierrez, Ralph L. Sacco, Tatjana Rundek

**Affiliations:** ^1^Department of Neurology, The Evelyn McKnight Brain Institute, Miller School of Medicine, University of Miami, Miami, FL, United States; ^2^Department of Systems Medicine, School of Medicine, University of Rome Tor Vergata, Rome, Italy; ^3^Department of Human Sciences and Quality of Life Promotion, San Raffaele Roma Open University, Rome, Italy; ^4^Department of Neurobiology, Karolinska Institute, Care Sciences and Society, Stockholm, Sweden; ^5^Department of Neurology, Vagelos College of Physicians and Surgeons and Mailman School of Public Health, Columbia University, New York, NY, United States

**Keywords:** carotid artery, plaque, atherosclerosis, ultrasonology, gray-scale median, vascular risk factors, glucose, lipids

## Abstract

Low Gray-Scale Median (GSM) index is an ultrasonographic parameter of soft, lipid rich plaque morphology that has been associated with stroke and cardiovascular disease (CVD). We sought to explore the contribution of the modifiable and not-modifiable cardiovascular risk factors (RFs) to vulnerable plaque morphology measured by the low GSM index. A total of 1,030 stroke-free community dwelling individuals with carotid plaques present (mean age, 71.8 ± 9.1; 58% women; 56% Hispanic, 20% Non-Hispanic Black, 22% Non-Hispanic White) were assessed for minimum GSM (min GSM) using high-resolution B-mode carotid ultrasound. Multiple linear regression models were used to evaluate the association between RFs and minGSM after adjusting for sociodemographic characteristics. Within an individual, median plaque number was 2 (IQR: 1–3) and mean plaque number 2.3 (SD: 1.4). Mean minGSM was 78.4 ± 28.7 (IQR: 56–96), 76.3 ± 28.8 in men and 80 ± 28.5 in women; 78.7 ± 29.3 in Hispanics participants, 78.5 ± 27.2 in Non-Hispanic Black participants, and 78.2 ± 29 in Non-Hispanic white participants. In multivariable adjusted model, male sex (β = −5.78, *p* = 0.007), obesity BMI (β = −6.92, *p* = 0.01), and greater levels of fasting glucose (β = −8.02, *p* = 0.02) and LDL dyslipidemia (β = −6.64, *p* = 0.005) were positively associated with lower minGSM, while presence of glucose lowering medication resulted in a significant inverse association (β = 7.68, *p* = 0.04). Interaction (with *p* for interaction <0.1) and stratification analyses showed that effect of age on minGSM was stronger in men (β = −0.44, *p* = 0.03) than in women (β = −0.20, *p* = 0.18), and in individuals not taking glucose lowering medication (β = −0.33, *p* = 0.009). Our study has demonstrated an important contribution of glycemic and lipid metabolism to vulnerable, low density or echolucent plaque morphology, especially among men and suggested that use of glucose lowering medication was associated with more fibrose-stable plaque phenotype (greater GSM). Further research is needed to evaluate effects of medical therapies in individuals with vulnerable, low density, non-stenotic carotid plaques and how these effects translate to prevention of cerebrovascular disease.

## Introduction

Carotid plaque assessed by high-resolution ultrasonography is a well-validated marker of atherosclerosis and risk of stroke (1). Plaque densitometry, measured by the ultrasonographic gray-scale median (GSM) index, is a parameter of plaque morphology and a helpful predictor of stroke and its outcomes (2, 3). The GSM index represents a marker of plaque vulnerability with the potential clinical use because of its simplicity and reliability of assessment, low cost, and ability to be measured from plaque images collected from a clinical B-mode ultrasonography (2). Low GSM plaque values correspond to soft, echolucent plaque with high lipid content and a thin fibrous cap, whereas high GSM index represents echodense plaques with high fibrous content and calcification (3, 4). Low GSM values have been associated with higher prevalence of symptomatic carotid stenosis, neurological symptoms (2), and cerebrovascular disease (5). Recently, lower GSM values, established by brain magnetic resonance diffusion-weighted imaging (DWI), was found helpful to predict new cerebral ischemic lesions after carotid endarterectomy (6). We previously reported on the impacts of traditional and less traditional vascular risk factors on atherosclerotic plaque phenotypes, including plaque area and densitometry (7). In the Prospective Investigation of the Vasculature in Uppsala Seniors (PIVUS) study, the low levels of high-density lipoproteins (HDL) cholesterol, increased body mass index (BMI), and decreased glutathione levels were associated with the echolucent carotid plaque, implying the role of metabolic factors in plaque composition (8). However, not all studies were consistent and some reported no association between risk factors and grayscale ultrasonographic plaque features in middle-aged adults free of known cardiovascular disease (9). Therefore, we sought to investigate contribution of vascular risk factors to the vulnerable plaque morphology measured by the low GSM index in an urban, multi-ethnic cohort.

## Materials and Methods

### Study Populations

The Northern Manhattan Study (NOMAS) is an ongoing population-based study aimed to determine the incidence of stroke, cognitive decline, and the role of novel risk factors in a race/ethnically diverse community (10). The details of the NOMAS design have been described previously (11). Data were collected through interviews using standardized collection instruments, review of the medical records, and physical and neurological examinations (11). NOMAS was approved by the Institutional Review Boards of Columbia University Medical Center and the University of Miami. All participants gave written informed consent for participation in the study NOMAS subjects received carotid ultrasound at the time of baseline enrollment from 1999. There were no specific selection criteria for the participation in the carotid ancillary study. A sample of 1,790 stroke-free subjects has been enrolled into the NOMAS carotid ultrasound imaging substudy (1).

### Vascular Risk Factors

Definitions of vascular risk factors in NOMAS were described previously (11). In brief, race/ethnicity was self-identified based on questions adapted from the 2000 US census and classified into four categories (White non-Hispanic, Black non-Hispanic, Hispanic, and non-Hispanic other race). Hypertension was defined as a SBP ≥ 140 mm Hg or a DBP ≥ 90 mm Hg or a patient's self-report of a history of hypertension or use of antihypertensive medications. Diabetes was defined as fasting blood glucose ≥ 126 mg/dL or the patient's self-report of such a history or use of insulin or hypoglycemic medications. Dyslipidemia was defined as total cholesterol >200 mg/dL or self-reported history of increased blood cholesterol levels or cholesterol-lowering medication use. We did not capture duration of comorbidities before enrollment to NOMAS. Therefore, if present diabetes and dyslipidemia at baseline of NOMAS enrollment, they would have been present for at least 6–8 years before the start of ultrasound ancillary study. Medication for these specific diseases in NOMAS are classified in specific classes of medications (e.g., for diabetes, insulin, and oral glucose lowering med; for dyslipidemia statin, fibrates, for hypertension, ACE inhibitors/ARBS, Ca^2+^ channel blockers, diuretics, and beta-blockers). Cigarette smoking was categorized as non-smoker, former, or current smoker (within the last year) and pack-years of smoking were calculated. Mild to moderate alcohol use was defined as current drinking of >1 drink per month and ≤2 drinks per day. Body mass index (BMI) was calculated in kg/m^2^. Physical activity was defined as the frequency and duration of 14 different recreational activities during the 2-week period before the interview. Years of education were collected, and completion of high school was a proxy for socioeconomic status. Medical insurance status (Medicare or private insurance vs. Medicaid or uninsured) was used as a proxy of socioeconomic status.

### Carotid Ultrasound

High-resolution B-mode carotid ultrasound (GE LogIQ 700, 9–13-MHz linear-array transducer) was performed by trained and certified sonographers as previously detailed (12). The left and right carotid bifurcations and the internal and common carotid arteries were examined for the presence of plaque. Plaque was defined as an area of focal wall thickening 50% greater than surrounding wall thickness confirmed by marking and comparing plaque thickness with the thickness of the surrounding wall during scanning by electronic calipers (7). After image normalization using linear scaling, GSM of an operator-selected blood region inside the vessel lumen was mapped to 0 and the brightest region of the adventitia was mapped to 255 using M'Ath (Paris, France) (13). Both of these reference regions were ~0.4 mm2 in area and were selected on the first image of the image sequence. The reference GSM values calculated on the first frame were applied to that and all subsequent images. GSM was expressed for each plaque. The minimal GSM (minGSM) values of all carotid plaques insonated within an individual were averaged and considered a measure of echolucent or vulnerable plaque morphology (14).

### Statistical Analysis

Among a total of 3,298 subjects enrolled in NOMAS, 1,790 stroke-free subjects represent the sample size needed to reach a significant α level. Sample characteristics were summarized as means with standard deviation (SD) for continuous variables and reported as frequencies with percentages for categorical variables. Student *t*-test, or *F*-test when more than two groups, was used to compare group mean differences in minGSM, whereas Pearson correlation analyses were conducted to examine the correlation between minGSM and each continuous variable. A multiple linear regression model fully adjusted for sociodemographic was constructed to evaluate association of risk factors with minGSM and collinearity was evaluated using variance inflation factor (VIF). As a secondary and sensitivity analysis, a stepwise linear regression was performed to identify risk factors associated with minGSM independently. Two-way interactions between the significant factors were also conducted by inclusions of interaction terms of them in the regression models and stratification analyses were followed if the interactions with a *p* < 0.10. All analyses were done using SAS version 9.4 (SAS Institute, Cary, N.C.).

## Results

**Figure 1:**
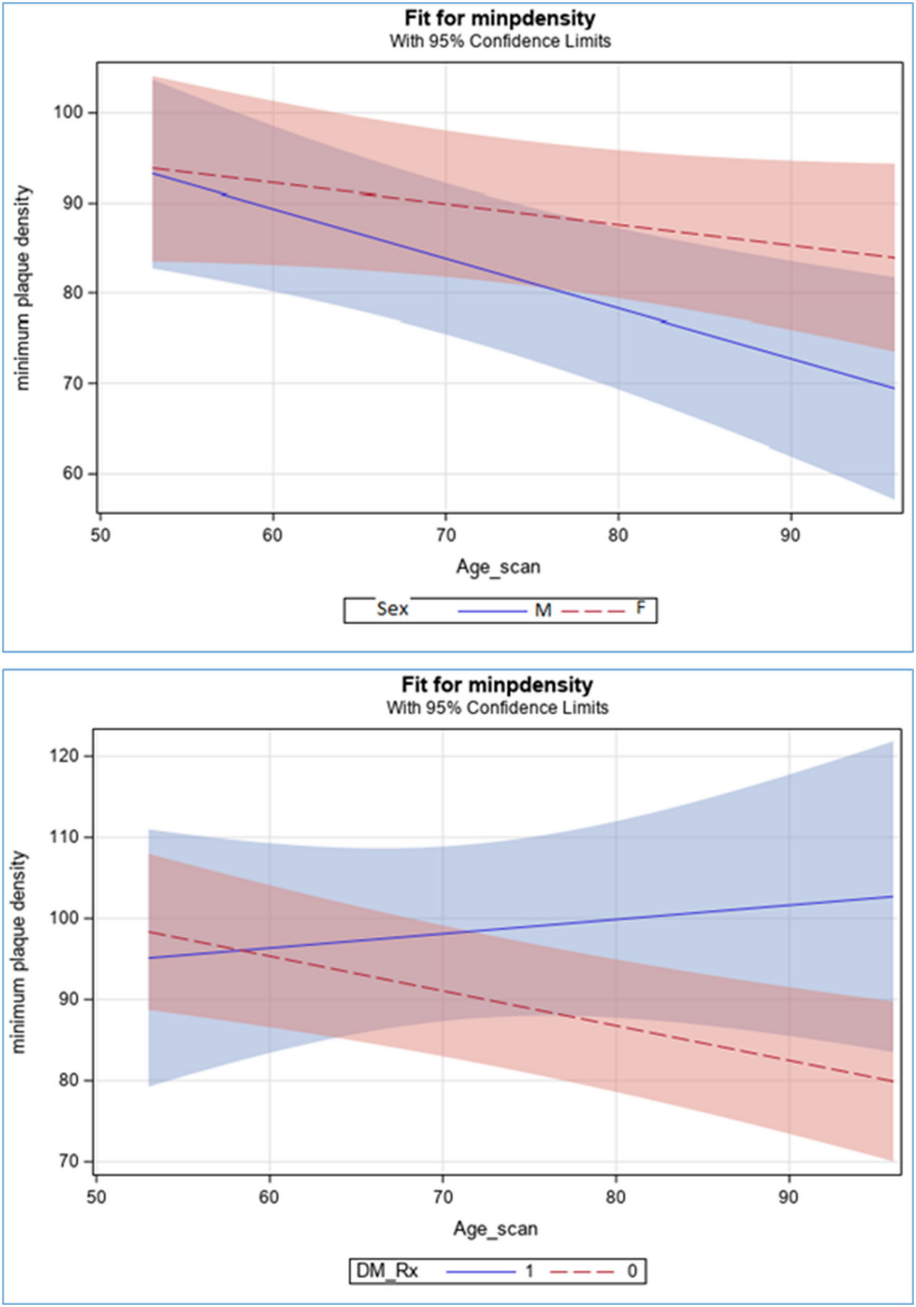
Effect of age on minGSM by sex and antidiabetic medications.

Among 1,790 stroke-free subjects, 1,030 subjects had at least one carotid plaque. Plaques were non-stenotic (<1% of subjects had carotid stenosis >50% on carotid ultrasound). The associations between demographic and clinical characteristics with minGSM are shown in Table 1. The mean age in the whole sample was 71.8 ± 9.1years; 58% were women; 56% Caribbean Hispanics, 20% Non-Hispanic Black, and 22% Non-Hispanic White. Mean minGSM was 78.4 ± 28.7 in all subjects (IQR: 56–96), 76.3 ± 28.8 in men and 80 ± 28.5 in women; 78.7 ± 29.3 in Hispanic, 78.5 ± 27.2 in Non-Hispanic black, and 78.2 ± 29 in Non-Hispanic white participants. In univariate analysis, male sex (*p* = 0.04), increased BMI ≥ 30 (*p* = 0.005) and fasting glucose level (*p* = 0.02) were significantly and inversely associated with minGSM, while lower HDL cholesterol (*p* = 0.05) was positively associated with minGSM (Table 1). In the fully adjusted model, minGSM-correlates were observed for age (β = −0.42, *p* = 0.001), male sex (β = −5.78, *p* = 0.007), BMI ≥ 30 (β = −6.29, *p* = 0.01), diabetes (β = −8.02, *p* = 0.02), dyslipidemia (β = −6.64, *p* = 0.005), and lipid lowering medication use (β = 7.68, *p* = 0.04). All factors had a VIF <3.5, suggesting that there was no high correlation between these factors (Table 2). Similarly, in multivariable adjusted model with stepwise selection, age, male sex, current smoking, obesity, diabetes, dyslipidemia, and antidiabetic medication use, were significantly associated with minGSM (Supplementary Table 1). Interaction (with *p* for interaction < 0.1) and stratification analyses showed that effect of age on minGSM was stronger in men (β = −0.44, *p* = 0.03) than in women (β = −0.20, *p* = 0.18), and in individuals without taking glucose lowering medication (β = −0.33, *p* = 0.009; Table 3; Figure 1).

**Table 1:** Demographic and clinical characteristic of study sample.

**Characteristics**	**Sample**		**Min. GSM**	***P*** **-value**
	***N***	**%**	**Mean ±SD**	
All	1,030	100%	78.4 ± 28.7	
**Sex**				0.04
Female	598	58%	80.0 ± 28.5	
Male	432	42%	76.3 ± 28.8	
**Race/ethnicity**				
Non-Hispanic White	223	22%	78.2 ± 29.0	**Ref**
Non-Hispanic Black	211	20%	78.5 ± 27.2	0.995
Hispanic	575	56%	78.7 ± 29.3	0.852
Non-Hispanic other	21	2%	74.1 ± 22.6	0.454
**High school completion**				0.77
No	515	50%	78.2 ± 28.8	
Yes	515	50%	78.7 ± 28.5	
**Private insurance/medicare**				0.89
No	234	23%	78.2 ± 30.1	
Yes	796	77%	78.5 ± 28.3	
**Moderate alcohol drinking**				0.89
No	614	60%	78.3 ± 28.0	
Yes	416	40%	78.6 ± 29.6	
**Physical activity**				0.46
No	444	43%	77.7 ± 28.5	
Yes	581	56%	79.0 ± 28.9	
**Smoking**				
Never	444	43%		**Ref**
Former	399	33%		0.983
Current	187	69%		0.048
**BMI, Kg/m** ^**2**^				
<25	278	27%	81.3 ± 29.6	**Ref**
25–29	464	45%	78.7 ± 28.5	0.18
≥30	86	28%	75.1 ± 27.8	0.005
**BS, mg/dL**				
<100	662	66%	79.3 ± 29	**Ref**
100–125	165	17%	79.6 ± 28.6	0.909
>125	170	17%	74.2 ± 27.1	0.036
**SBP, mmHg**				
<120	127	12%	79.8 ± 29.6	**Ref**
120–139	313	30%	78.9 ± 28.2	0.808
≥140	589	57%	77.9 ± 28.7	0.636
**DBP, mmHg**				
<80	508	49%	79.8 ± 29	**Ref**
80–89	224	22%	76.2 ± 27.5	0.081
≥90	297	29%	77.7 ± 29	0.224
**LDL, mg/dL**				
<130	512	52%	79.4 ± 29.6	**Ref**
130–149	205	21%	79.2 ± 28.5	0.867
≥150	276	28%	75.7 ± 27.4	0.088
**HDL, mg/dL**				
≥40 for M, ≥50 for f	473	47%	78.2 ± 28.9	**Ref**
≥30 for M, ≥40 for f	338	34%	79.3 ± 27.2	0.725
<30 for M, <40 for f	191	19%	77.2 ± 31.2	0.586
**TC, mg/dL**				
<200	478	48%	77.4 ± 29.6	**Ref**
200–239	351	35%	80 ± 28.2	0.171
**Antihypertension medication**				0.35
No	576	56%	79.2 ± 29.8	
Yes	454	44%	77.5 ± 27.2	
**Lipid-lowering medication**				0.25
No	839	81%	78.9 ± 28.8	
Yes	191	19%	76.3 ± 28.3	
**Glucose-lowering medication**				0.90
No	870	84%	78.5 ± 28.8	
Yes	160	16%	78.2 ± 27.8	
**Hypertension**				0.88
No	282	27%	78.5 ± 29.0	
Yes	748	73%	78.4 ± 28.5	
**Hypercholesterolemia**				0.19
No	690	67%	79.3 ± 28.9	
Yes	340	33%	76.8 ± 28.1	
**Diabetes**				0.09
No	798	77%	79.2 ± 28.8	
Yes	232	23%	75.8 ± 28.0	
	**Mean**	**SD**	**Correlation**	***P*** **-value**
Age, years	71.8	9.1	−0.049	0.11
BMI, Kg/m^2^	28.0	4.9	−0.068	0.03
SBP, mmHg	143.8	20.4	−0.043	0.17
DBP, mmHg	82.8	10.8	−0.053	0.09
Fasting Glucose, mg/dL	104.6	43.3	−0.075	0.02
LDL, mg/dL	129.7	36.4	−0.038	0.23
HDL, mg/dL	46.5	14.4	0.061	0.05

**Table 2:** Association of demographic and categorical vascular risk factors with min GSM.

**Variable**	**Beta**	**SE**	***P*** **-value**	**VIF**
**Age**	**−0.42**	**0.13**	**0.001**	**1.42**
**Male sex**	**−5.78**	**2.15**	**0.007**	**1.23**
Hispanic vs. non-Hispanic white	2.15	3.02	0.478	2.47
non-Hispanic black vs. non-Hispanic white	−0.91	3.10	0.768	1.75
Non-Hispanic other vs. non-Hispanic white	−3.38	6.77	0.618	1.12
High school completion (yes vs. no)	−0.61	2.40	0.800	1.57
Private insurance/medicare (yes vs. no)	4.92	2.57	0.056	1.28
Smoker current vs. never smoker	−4.68	2.83	0.098	1.29
Smoker former vs. never smoker	2.62	2.05	0.236	1.27
Moderate alcohol drinking (yes vs. no)	0.32	2.05	0.877	1.08
Physical activity (yes vs. no)	1.62	2.01	0.419	1.07
BMI overweight	−2.72	2.42	0.263	1.59
**BMI obese**	**−6.92**	**2.84**	**0.015**	**1.72**
Fasting glucose border	0.46	0.67	0.862	1.10
**Fasting glucose diabetic**	**−8.02**	**3.64**	**0.028**	**1.20**
SBP border	−0.74	3.45	0.830	2.68
SBP hypertension	0.49	3.48	0.888	3.20
DBP border	−2.89	2.63	0.272	1.28
DBP hypertension	−2.76	2.55	0.280	1.53
LDL border	−2.19	2.56	0.394	1.19
**LDL dyslipidemia**	**−6.64**	**2.34**	**0.005**	**1.22**
HDL border	2.26	2.32	0.330	1.35
HDL dyslipidemia	0.19	2.94	0.949	1.42
**Glucose-lowering medication (yes vs. no)**	**7.68**	**3.70**	**0.038**	**1.91**
Antihypertension medication (yes vs. no)	−0.80	2.15	0.709	1.23
Lipid-lowering medication (yes vs. no)	−2.51	2.73	0.359	1.12

*Fully adjusted model: age, sex, race/ethnicity, high school completion and vascular risk factors (moderate alcohol use, moderate-heavy physical activity, BMI, systolic blood pressure, diastolic blood pressure, anti-hypertensive medication use, diabetes, LDL, HDL, cholesterol-lowering medication use). VIF, variance inflation factor. The bold values are significant, with a p-value < 0.05*.

**Table 3:** Effect of age on minGSM by sex and antidiabetic medication use and their interactions.

**Variable**	**Beta**	**SE**	***P*** **-value**	***p*** **for interaction**
**Sex**
Male	−0.44	0.18	0.02	0.04
Female	−0.20	0.15	0.18	
**Glucose-lowering use**
Yes	0.23	0.33	0.48	0.07
No	−0.33	0.12	0.009	

## Discussion

In this study, we reported significant associations between glycemic and lipidic parameters with unfavorable carotid plaque morphology measured by the ultrasonographic GSM index. Our results suggest that along age and male sex, increased levels of fasting glucose, LDL cholesterol, and greater BMI are particularly critical for vulnerable plaque morphology, while glucose lowering medication use was protective. Moreover, the effects of these factors were more pronounced in older men than in older women, and in older patients treated for diabetes. No differences in vulnerable plaque morphology or the effects of risk factors on plaque morphology were noted between race-ethnic groups of participants. Our results indicate an atherosclerotic plaque phenotype that may explain a greater prevalence of extracranial atherosclerotic stroke in men than in women. Smoking did not affect echolucent plaque phenotype in our study. Aggressive treatments of metabolic factors and glycemic control provide opportunities for effective prevention of stroke and other atherosclerotic events. The carotid GSM index may be utilized as an effective non-invasive imaging biomarker to monitor vulnerable atherosclerotic plaque morphology and success of preventive interventions.

The relevance of low GSM values in carotid plaque has been established in a meta-analysis including 7,557 subjects with a mean follow-up of 37.2 months, where echolucent carotid plaques were associated with an increased risk of ipsilateral stroke regardless of the degree of stenosis (15). Moreover, in the Imaging in Carotid Angioplasty and Risk of Stroke (ICAROS) study, lower GSM was associated with poor outcome after intervention and low GSM improved stratification of patients for carotid endarterectomy or stenting (16). Lowest GSM has been characterized with a presence of lipid core, inflammation, neovascularity, and foam cells (17). Age and male sex are the main risk factors for the vulnerable atherosclerotic plaque morphology. In Evaluation of Rosuvastatin (METEOR) study, older age (mean 84 ± 29) was associated with more echolucent plaques (18). Age-related changes in arterial hemodynamic and increased arterial stiffness lead to an increase prevalence of atheromatous plaques and decrease in fibrous plaques morphology, characterized by higher macrophage and less smooth muscle cells content. This process seems to be accelerated in men, as suggested in our study as well as in the Tromsø Study (19). In patients with recent ischemic event, older men had carotid plaque with lower GSM values compared to women of same age (20), consistent with our findings. Sex seems to be a critical determinant of atherogenic lipoprotein levels with glycemic control and LDL cholesterol playing a central role (21). Glycated LDL after oxidation increases their permeation in the endothelial space generating atherosclerotic process, especially in a state of vascular inflammation that is higher in older men than women (20). Moreover, a histopathological study has demonstrated that atherosclerotic carotid plaques obtained from men had a greater prevalence of plaque hemorrhage and more vascular inflammation (22). Here, we extend these observations to a large multi-ethnic stroke-free population.

Alteration in glycemic metabolism and dyslipidemia plays an important role in the development of heterogeneous plaque morphology. They are directly related to change in BMI that is considered an independent risk factor for carotid plaque destabilization (23). In the Atherosclerosis Risk in Young Adults (ARYA) Study, high BMI was associated with lower GSM values independently of other RFs and phenotypes of atherosclerosis (24). Hyperglycemia and high LDL cholesterol levels change the structure of plaque to rise its susceptibility to ulceration and to become more prone to rupture and consequently cause embolic vascular events. We previously reported that this unfavorable plaque morphology can be reversed by reducing the levels of LDL cholesterol using a high-dose atorvastatin intervention in 30 days (25).

The association between low GSM and type 2 diabetes has been established (26). A combined analysis of 5 longitudinal studies with a total of 3,263 patients with uncontrolled diabetes but without apparent CVD demonstrated that presence of low-GSM echolucent plaques at baseline were the most powerful prognostic factor for the occurrence of CVD, even after adjustment for traditional risk factors (9). In our study, there was a significant association with fasting glucose even in those without diabetes, and a protective effect of lipid lowering medication especially among older patients. These evidences indicate that increased levels of glucose may trigger the mechanisms leading to echolucent plaque, which can be reverted by reducing glucose levels. Soft plaques are more present in diabetic patients and higher levels of glycated hemoglobin (HbA1c), further suggesting the role of glucose homeostasis in the development of unstable plaques (27).

Recently, in The Multi-Ethnic Study of Atherosclerosis (MESA), total plaque area, but not grayscale plaque features, was associated with risk factors and predicted incident coronary heart disease events (9). However, significant relationships with risk factors were observed after adjustment for Non-Hispanic Black (vs. Non-Hispanic White participants) who had plaques with the lower GSM values (9). Discrepancy between our study and MESA are mostly due to the differences in study designs, inclusion criteria, and ultrasound methods and definition of atherosclerosis.

Limitations of our study need to be acknowledged. The cross-sectional nature of the current findings does not allow inference of temporal effects or causality. Our study mainly included well-known atherosclerotic risk factors, whereas other factors of possible importance for atherosclerosis such as diet or endothelial function were not considered. Moreover, GSM analysis represents a mean value of whole atherosclerotic area and does not reflect the presence of particular regional plaque components. However, a lack of regional plaque analyses may have underestimated plaque vulnerability and therefore attenuated true associations. The major strengths of our study include a well-characterized multi-ethnic population representative of an urban community and standardized and carful assessments of carotid plaque presence and echogenic morphology.

In conclusion, it is important to highlight the usefulness of GSM analysis as ultrasound markers in the clinical practice, since based on its low cost, and lack of radiation can be repeated routinely in diabetic and dyslipidemic patients to evaluate, non-invasively, the risk for vascular diseases. By understanding the impact of metabolic risk factors, such as increased levels of lipids and glycemia, on high-risk plaque morphology in multi-ethnic communities is of great importance for intensive interventions aimed at reversal of unfavorable plaque morphology and successful prevention of stroke and cardiovascular disease.

## Data Availability Statement

The original contributions presented in the study are included in the article/Supplementary Material, further inquiries can be directed to the corresponding author/s.

## Ethics Statement

The studies involving human participants were reviewed and approved by Institutional Review Boards of Columbia University Medical Center and the University of Miami. The patients/participants provided their written informed consent to participate in this study.

## Author Contributions

DD-M, CD, MC, and TR: conceptualization, validation, data curation, and project administration. DD-M, CD, and MC: writing—original draft. CD: formal analysis. ME, JG, RS, and TR: writing—review and editing. DD-M and TR: funding acquisition. All authors have assisted with manuscript preparation and approved the final manuscript.

## Funding

This work was supported by the National Institute of Neurologic Disorders and Stroke Grants: R01NS040807 and R0129993, and the Evelyn F. McKnight Brain Institute.

## Conflict of Interest

The authors declare that the research was conducted in the absence of any commercial or financial relationships that could be construed as a potential conflict of interest.

## Publisher's Note

All claims expressed in this article are solely those of the authors and do not necessarily represent those of their affiliated organizations, or those of the publisher, the editors and the reviewers. Any product that may be evaluated in this article, or claim that may be made by its manufacturer, is not guaranteed or endorsed by the publisher.
