# Supplementary material for: Association of Carotid Plaque Morphology and Glycemic and Lipid Parameters in the Northern Manhattan Study
Source: Front Cardiovasc Med. 2022 Jan 24;9:793755. doi: 10.3389/fcvm.2022.793755 (PMC8818735; doi:10.3389/fcvm.2022.793755)
Supplement: Supplementary file 1 [file Table_1.DOCX]

Supplementary Material

| **Supplemental Table 1. Factors Associated with Min GSM Based on Stepwise Linear Regression** | | | | | |
| --- | --- | --- | --- | --- | --- |
| **Variable** | **Beta** | **SE** | **95% CI** | | **P-value** |
| Age | -0.32 | 0.11 | -0.53 | -0.10 | 0.004 |
| Male sex vs. Female | -5.51 | 2.00 | -9.43 | -1.59 | 0.006 |
| Current Smoker | -5.56 | 2.49 | -10.45 | -0.68 | 0.026 |
| BMI ≥30 Kg/m^2^ | -5.33 | 2.19 | -9.62 | -1.03 | 0.015 |
| Fasting glucose ≥ 126 mg/dL | -8.07 | 3.42 | -14.77 | -1.37 | 0.018 |
| LDL ≥ 150 mg/dL | -5.63 | 2.13 | -9.77 | -1.44 | 0.008 |
| Antidiabetic medication use | 7.21 | 3.53 | 0.29 | 14.14 | 0.041 |
